# TB-DLossNet: Fine-Grained Segmentation of Tea Leaf Diseases Based on Semantic-Visual Fusion

**DOI:** 10.3390/plants15071035

**Published:** 2026-03-27

**Authors:** Shuqi Zheng, Hao Zhou, Ziyang Shi, Fulin Su, Wei Shi, Ruifeng Liu, Lin Li, Fangying Wan

**Affiliations:** 1 School of Electronic Information and Physics, Central South University of Forestry and Technology, Changsha 410004, China; 20233409@csuft.edu.cn (S.Z.); 20231100403@csuft.edu.cn (H.Z.); 20231200582@csuft.edu.cn (Z.S.); 20241200786@csuft.edu.cn (F.S.); 20221200478@csuft.edu.cn (W.S.); t19940562@csuft.edu.cn (F.W.); 2 College of Forestry, Central South University of Forestry and Technology, Changsha 410004, China

**Keywords:** deep learning, *Camellia oleifera*, VMamba, multimodal integration, pest and disease segmentation

## Abstract

*Camellia oleifera* is an economically vital woody oil crop. Its productivity and oil quality are severely compromised by various diseases. Implementing pixel-level lesion segmentation within complex field environments is crucial for advancing precision plant protection. Despite recent progress, existing segmentation methods struggle with three primary challenges: semantic ambiguity arising from evolving pathological stages, blurred boundaries due to overlapping lesions, and the high omission rate of micro-lesions. To address these issues, this paper presents TB-DLossNet (Text-Conditioned Boundary-Aware Network with Dynamic Loss Reweighting), a novel segmentation framework based on semantic-visual multi-modal fusion. Leveraging VMamba as the visual backbone, the proposed model innovatively integrates BERT-encoded structured text as an auxiliary modality to resolve visual ambiguities through cross-modal semantic guidance. Furthermore, a boundary enhancement branch is incorporated alongside a multi-scale deep supervision strategy to mitigate boundary displacement and ensure the topological continuity of lesion structures. To tackle the detection of small-scale targets, we designed a dynamic weight loss function conditioned on lesion area, significantly bolstering the model’s sensitivity to minute pathological features. Additionally, to alleviate the scarcity of high-quality data, we curated a comprehensive multi-modal dataset encompassing seven typical diseases of *Camellia oleifera*. Experimental results demonstrate that TB-DLossNet achieves a Mean Intersection over Union (mIoU) of 87.02%, outperforming the state-of-the-art unimodal VMamba and multimodal Lvit by 4.9% and 2.59%, respectively. Qualitative evaluations confirm that our model exhibits lower false-negative rates and superior boundary-fitting precision in heterogeneous field scenarios. Finally, generalization tests on an apple disease dataset further validate the robustness and transferability of the proposed framework.

## 1. Introduction

*Camellia oleifera*, a premier woody oilseed species predominantly cultivated in the hilly regions of Southern China and introduced to climatically analogous zones in Southeast Asia, possesses profound economic and ecological significance. Its seed oil, colloquially heralded as “Oriental Olive Oil”, has been clinically linked to a reduced risk of major cardiovascular events [1]. Currently, China’s cultivation area for *Camellia oleifera* exceeds 75 million mu (approx. 5 million hectares), yielding approximately 1.1 million tons of tea oil—representing 90–95% of global production [2]. Bolstered by international demand and evolving trade corridors, exports reached 52,949 kg in 2024 [3]. Furthermore, industrial chain integration has enabled the high-value extraction of active compounds such as tea saponins, polysaccharides, and polyphenols for applications in chemical engineering, animal feed, and materials science [4].

Despite its economic potential, *Camellia oleifera* productivity is severely constrained by biotic stresses. Disease outbreaks not only compromise oil quality but also trigger significant flower and fruit abscission [5]. Specifically, Anthracnose causes expanding necrotic lesions alongside bud drop and shoot blight, potentially leading to systemic decline or tree mortality. This disease typically incurs annual yield losses of 10–30%, escalating to 40–80% in high-incidence regions [6,7]. Similarly, Soft Rot directly impacts marketable yield through fruit decay, accounting for losses between 10% and 45% [8,9]. Consequently, robust disease detection has become a fundamental prerequisite for ensuring yield stability and quality control.

In practice, disease monitoring still predominantly necessitates manual field inspections and visual diagnosis. However, consensus in the literature highlights that such approaches are inherently labor-intensive, inefficient, and lack the consistency required for large-scale or high-frequency monitoring tasks [10]. The early automatic recognition of visible light images relied on handmade features, including color thresholds, texture descriptors and morphological orators. Although these methods can work normally in a controlled environment, they are highly sensitive to changes in the scale, color and position of lesions, requiring a large number of parameter adjustments, and poor robustness in heterogeneous field environments [11,12].

Driven by advancements in data acquisition and computational power, machine learning has been progressively integrated into agricultural pathology [13]. Early learning-based frameworks, such as Support Vector Machines (SVM) and decision trees, depended on manual feature engineering. Although these mitigated some reliance on fixed thresholds, they remained limited by the shallow representational capacity of hand-crafted features, failing to encapsulate the complex morphological and textural diversity of lesions [14]. In order to overcome these limitations, deep learning has become the ultimate example of precise disease division in modern agriculture.

Research on deep learning-based semantic segmentation has proliferated within agricultural contexts, witnessing continuous refinement in pixel-level disease identification across diverse crop species. For instance, Pan et al. [15] leveraged PSPNet on UAV-acquired imagery to differentiate healthy wheat, infected areas, and bare soil. Similarly, targeting grapevine canopies, Li et al. [16] developed an enhanced U-Net to isolate valid leaf ROIs and localize pathological regions, thereby improving diagnostic precision in complex backgrounds. Furthermore, Zang et al. [17] introduced RSE-Swin Unet to bolster feature representation for wheat powdery mildew segmentation. Beyond agriculture, deep learning has also demonstrated strong performance in other complex detection tasks, such as industrial fault detection, where neural networks have been used to identify subtle internal leaks in hydraulic actuator cylinders [18]. These milestones emphasize the effectiveness of deep learning in automated disease extraction and provide a technical basis for subsequent quantitative analysis and precise management.

Recently, architectural innovations—particularly those integrating multi-branch structures and sophisticated context modeling—have significantly advanced the segmentation of coexisting and micro-scale lesions. A notable breakthrough is the Mamba architecture [19], which utilizes Selective State Space Models (SSMs) to achieve long-range dependency modeling and global context aggregation, offering a computationally efficient alternative to the self-attention paradigm. Building upon this, VMamba introduced a 2D selective scanning (SS2D) mechanism to traverse feature maps in four directions [20]. This approach captures exhaustive global dependencies while maintaining near-linear computational complexity relative to image resolution. Consequently, VMamba has demonstrated superior performance over established backbones like Swin and ConvNeXt across ImageNet, COCO, and ADE20K benchmarks, particularly in terms of throughput and high-resolution scalability.

Despite the strengths of these multi-scale context-aware models, their reliance on a single visual modality (typically RGB) presents inherent limitations for *Camellia oleifera* disease analysis. Specifically, micro-lesions characterized by subtle visual features and low pixel occupancy are frequently overshadowed by dominant background classes during training, leading to significant omission rates. In addition, in the case of coexistence of diseases, the interclass similarity of color and texture often leads to semantic misclassification. Most importantly, traditional models prioritize regional semantic consistency over fine-grained structural integrity, which often leads to boundary displacement and topological fragmentation. Although recent efforts have begun addressing scale variance and boundary ambiguity through multi-scale aggregation [21], the challenge remains: a single modality is fundamentally insufficient to resolve the intricate requirements of small-lesion detection, multi-disease discrimination, and precise boundary refinement in heterogeneous field environments.

Given the absence of semantic priors, the low occupancy of lesion pixels, and the susceptibility of boundary details to degradation, unimodal visual information frequently proves inadequate in addressing the multifaceted challenges of disease segmentation. Consequently, multimodal fusion has emerged as a promising paradigm. By integrating auxiliary modalities such as textual descriptions and meteorological data [22,23], models can derive enriched representations and capture subtle nuances elusive to a single modality, thereby enhancing robustness and precision—particularly for early-stage lesions and in heterogeneous backgrounds. Specifically, text data is easier to access and expand than labor-intensive pixel annotations. It provides key semantic a priori of symptom clues, anatomical location and pathological staging. These linguistic cues complement visual features—such as texture, color, and morphology—imposing semantic constraints that mitigate inter-class ambiguity and misclassification of visually similar pathologies [24]. However, most existing agricultural data sets are mainly image-centered and lack descriptions or environmental background consistent with experts. This defect prevents the model from making full use of the semantic trajectory of disease progression. In order to make up for this gap, this study incorporates text a priori into the segmentation framework to improve the location and segmentation of lesions under low contrast and complex on-site conditions.

In summary, despite the advancements of deep learning in *Camellia oleifera* disease analysis, several formidable challenges persist:

1. **Semantic Ambiguity and Localization Bias:** In authentic field environments, lesion positioning is stochastic and visual signatures evolve across pathological stages. Relying solely on visual features often leads to unstable localization and failure to differentiate disease stages, resulting in significant segmentation inaccuracies.
2. **Severe Class Imbalance:** A pronounced disparity in pixel distribution exists between lesion regions and background/leaf tissues. When micro-lesions exhibit low pixel occupancy or infrequent occurrence, the optimization process is often dominated by majority classes, thereby attenuating the model’s sensitivity and representational capacity for minority pathological features.
3. **Structural Fragmentation and Boundary Displacement:** When multi-scale targets coexist, conventional models prioritize region-level semantic consistency at the expense of boundary integrity and structural continuity. This manifests as over-smoothing or fragmented predictions. Furthermore, deep architectures without robust intermediate supervision are susceptible to training instability and feature degradation.
4. **Scarcity of Vision-Language Datasets:** There is a notable lack of comprehensive datasets that integrate aligned visual and linguistic information. While imagery provides essential visual cues, it remains insufficient for a holistic characterization of complex disease dynamics.

To address the aforementioned challenges, this paper proposed TB-DLossNet, a novel multimodal disease segmentation framework that synergistically integrates visual and textual information. By incorporating an area-adaptive dynamic loss, a boundary-aware branch, and multi-layer deep supervision, the framework effectively mitigates issues related to semantic prior deficiency, class imbalance, and boundary ambiguity, significantly enhancing segmentation precision in complex, low-contrast field environments. The primary contributions are summarized as follows:

**(1)**  **Multimodal Feature Fusion with Semantic Priors:** We propose a cross-modal fusion strategy that integrates a BERT-based textual encoder with a VMamba-based visual backbone. By leveraging structured text as a high-level semantic guide, the model resolves visual ambiguities in diseased regions, particularly when pathological signatures are subtle or visually indistinguishable from the background.
**(2)**  **Area-Adaptive Dynamic Loss Function:** To tackle severe class imbalance where micro-lesions are frequently overshadowed by dominant background pixels, we designed a dynamic loss function conditioned on lesion area. By assigning higher loss weights to smaller targets, this mechanism amplifies the gradient contribution of micro-scale features during optimization, effectively reducing false negatives and ensuring stable segmentation across diverse scales.
**(3)**  **Boundary-Aware Refinement and Multi-Scale Deep Supervision:** We introduce a dedicated boundary-aware branch coupled with a multi-scale side-output supervision strategy. The former imposes explicit geometric constraints to preserve structural integrity and sharp contours, while the latter reinforces gradient flow through intermediate layers, ensuring a robust training process and mitigating the risk of feature degradation in deep architectures.
**(4)**  **Curation of a Novel Multimodal Dataset for *Camellia oleifera* Diseases:** In collaboration with forestry experts, we have developed a comprehensive multimodal dataset encompassing seven typical *Camellia oleifera* diseases. Beyond pixel-level masks, the dataset provides fine-grained textual descriptions of pathological features—such as morphology, coloration, and spatial distribution patterns—thereby bridging the existing gap in multimodal agricultural data and offering a high-value resource for future research.

## 2. Related Work

The U-Net architecture [25] is one of the fundamental encoder-decoder frameworks in the area of semantic segmentation. It has been widely applied in region-of-interest extraction in medical and agricultural imaging by using skip connections to maintain fine-grained spatial information during contextual fusion. The next generation advancements have involved the development of multi-scale modeling such as DeepLabv3+ [26], which uses Atrous Spatial Pyramid Pooling (ASPP) to trade off the semantic depth with boundary detail. In order to overcome the shortcomings of different lesion shapes and complicated foliar textures, researchers have also introduced residual units and state-of-the-art skip connections (e.g., MU-Net) to improve the representation of features [27]. But these strictly vision-based schemes tend to make models interpret semantics merely on the basis of simple visual clues such as color and texture. This strategy fails both when pathological conditions show chromatic changes and when inter-class visual analogies cause confusion.

Recently, transformer-based architectures have revolutionized the field of semantic segmentation by leveraging self-attention mechanisms to achieve global context modeling. The evolution of these models follows a clear trajectory toward more efficient and versatile representations. Early pioneering work such as SETR [28] adopted a pure Vision Transformer (ViT) as the encoder to capture long-range dependencies from a sequence-to-sequence perspective, yet suffered from high computational costs and the loss of spatial details due to single-scale feature representations. To address these limitations, hierarchical Vision Transformers were introduced; Swin Transformer [29] proposed shifted windows to generate multi-scale feature pyramids with linear computational complexity, while SegFormer [30] designed a hierarchical structure without positional encodings, enabling robust performance across varying resolutions. Building upon these advances, unified transformer-based frameworks have emerged to handle multiple segmentation tasks within a single architecture. Mask2Former [31] unified semantic, instance, and panoptic segmentation through masked attention, which focuses on predicting mask queries within regions of interest, while OneFormer [32] further introduced task-conditioned decoding to enable universal segmentation without task-specific heads. Despite their strong generalization capability on large-scale natural image datasets, these transformer-based models face significant challenges when directly adapted to fine-grained agricultural lesion segmentation. High interclass visual similarity, subtle boundary transitions, and severe pixel-level imbalance between lesion and background regions often lead to suboptimal performance, as self-attention mechanisms tend to prioritize global statistical patterns over fine-grained pathological details.

To further incorporate high-level semantic priors beyond visual appearance, vision–language multimodal learning has emerged as a promising direction. A pioneering example is LSeg [33], which incorporates textual descriptions into a transformer-based segmentation model to enable language-driven semantic segmentation, demonstrating highly competitive zero-shot and few-shot results by aligning pixel embeddings with language embeddings. This paradigm has recently been extended to agricultural scenarios; LVR [34] used textual cueing to offer initial suggestions in rice disease segmentation, whereas ITIMCA [35] used CLIP based alignment to be more effective than any of the unimodal models in the case of cassava leaves. In spite of these achievements, the current multimodal systems essentially depend on traditional CNN or ViT structures and have generalized texts which do not always match well with organized field observations. In addition, efficient segmentation using multimodality demands a visual structure that can capture long-range interdependencies but still allow a steady flow of spatial details. It appears that the use of novel State Space Models (SSMs) like VMamba has not been well explored in agriculture multifunctional activities. In order to fill this research gap, the present paper proposes a structured text branch coded by BERT which is combined across different stages with VMamba visualization features. This architecture gives direct semantic direction especially when pathological signals are visually indistinct or have large temporal variation.

Besides the limitations of semantics, the challenges of class imbalance and the identification of micro-lesions have remained a constant challenge in agricultural disease segmentation. Lesion pixels generally represent a small proportion of a tissue compared to the number of pixels representing the background or leaves causing the training process to be dominated by majority classes and underrepresentation of small-scale pathological features. To address this, adaptive loss designs have been incorporated into different frameworks, e.g., the two-stage DeepLabv3+ framework used on the apple leaf lesion segmentation task [36]. The latest developments in vision-language fusion and adaptive weighting have also shown significant advantages. It is interesting to note that in the multimodal RLEM-Net of *Camellia oleifera* [37], the addition of the textual branch increased the mIoU by 1.29%, and the added value of the Reinforcement Learning Weight Adjuster (RLWA) by 2.47. This cumulative improvement of 4.85% has highlighted the effectiveness of sample-dependent weighting in distinguishing small lesions and difficult areas. Nevertheless, most of these weighting schemes tend to depend upon hard hyperparameters or global settings and do not respond to image-specific changes in the size of lesions. Therefore, this paper proposes a dynamic loss reduction mechanism where the loss is adaptively reweighted depending on the true lesion occupancy in pixels. Mechanistically, this will guarantee continuous maximization attention on micro-lesions, hence counteracting the negative impact of class imbalance on segmentation accuracy.

Regardless of improved regional predictions, agricultural segmentation is still vulnerable to boundary uncertainty and structural discontinuity. Pathological boundaries tend to be characterized by gradual shifting or environmental noise, which can cause contour displacement and over-smoothing. Explicit boundary constraints on general semantic segmentation tasks have always produced performance increases. As an example, Wang et al. [38] proposed an active boundary loss that encourages geometric alignment between predicted and ground-truth boundaries, improving boundary quality and mIoU in semantic segmentation. In addition, deep architectures tend to suffer in suboptimal gradient propagation and training instability when only trained at the last output. Quantification of the improvement is enabled by integrating multi-scale side-output (deep) supervision. Encouraged by these results, we integrate into our model a special boundary prediction branch to impose geometric constraints, along with multi-scale deep supervision to achieve joint optimization. In spite of the fact that the boundary branch focuses on displacement and missing details, the side-output supervision guarantees strong intermediate feature learning, and together helps to restore sharp and constant lesion structures in complex fields.

## 3. Results and Discussion

To ensure the reproducibility of our experimental findings, all evaluations were conducted on a standardized computing platform, thereby eliminating potential performance discrepancies attributable to hardware variations. The detailed hardware specifications, software environment, and experimental hyperparameters are tabulated in Table 1 and Table 2.

### 3.1. Evaluation Index

The proposed framework was evaluated using four standard metrics derived from the confusion matrix: Precision, Recall, F1-score and mean Intersection over Union (mIoU). Considering the image domain Ω, let G represent the ground truth region and $\hat{G}\;$ represent the predicted region. The definitions are as follows:

(1) $$\begin{array}{rrrr} TP: & \left\{x\in \Omega |y(x)=1\wedge \hat{y}(x)=1\right\} & TN: & \left\{x\in \Omega |y(x)=0\wedge \hat{y}(x)=0\right\}\\ FP: & \left\{x\in \Omega |y(x)=0\wedge \hat{y}(x)=1\right\} & FN: & \left\{x\in \Omega |y(x)=1\wedge \hat{y}(x)=0\right\} \end{array}$$

Here, True Positives (TP) indicate correctly identified disease regions; True Negatives (TN) denote healthy tissue areas accurately classified as non-diseased; False Positives (FP) correspond to healthy areas misclassified as diseased (over-detection errors); and False Negatives (FN) represent actual disease regions that the model failed to detect (missed detection errors).

Precision is defined as the ratio of correctly predicted disease pixels to all pixels predicted as diseased, as expressed in Equation (2).

(2) $$Precision=\frac{TP}{TP+FP}$$

Recall (or Sensitivity) measures the fraction of actual disease pixels that are correctly identified, as expressed in Equation (3).

(3) $$Recall=\frac{TP}{TP+FN}$$

Since Precision and Recall are often in tension, the F1-score is used as their composite metric. It is the harmonic mean of the two, which penalizes low values in either component more severely than the arithmetic mean:

(4) $$F1=\frac{2\cdot Precision\cdot Recall}{Precision+Recall}=\frac{2TP}{2TP+FP+FN}$$

Consequently, the F1-score provides a single summary statistic that balances the trade-off between false positives and false negatives.

The Intersection over Union (IoU) measures the spatial overlap between the predicted segmentation mask and its corresponding ground truth mask for a given class. For class i, the IoU is computed as shown in Equation (5).

(5) $${IoU}_{i}=\frac{\left|A_{i}\cap B_{i}\right|}{\left|A_{i}\cup B_{i}\right|}$$

The mean Intersection over Union (mIoU) serves as a comprehensive metric for evaluating segmentation accuracy by averaging the IoU values across all semantic categories, as formulated in Equation (6).

(6) $$mIoU=\frac{1}{N}\sum_{i=1}^{N}{IoU}_{i}$$

For the per-class IoU, we compute the metric from confusion matrix totals aggregated across the entire test set. This provides a more robust, global estimate of performance for each class. For a class c, the ${TP}_{c}=\sum_{i}T\;P_{i,c}$, ${FP}_{c}=\sum_{i}F\;P_{i,c}$, ${FN}_{c}=\sum_{i}F\;N_{i,c}$ are first accumulated over all N test images. The global IoU for class c is then given by:

(7) $${IoU}_{global}(c)=\frac{{TP}_{c}}{{TP}_{c}+{FP}_{c}+{FN}_{c}}$$

### 3.2. State-of-the-Art Comparison

To rigorously evaluate the performance of the proposed TB-DLossNet framework for *Camellia oleifera* leaf disease segmentation, we established a comprehensive benchmarking suite. This includes several representative unimodal semantic segmentation networks: VMamba, U-Net [39], PSPNet [40,41], HRNet [42], DeepLabv3+ [26], Mask2Former [31], and OneFormer [32]. Furthermore, LViT [43], a state-of-the-art image-text multimodal model, was introduced as a competitive multimodal baseline.

**Figure 1:**
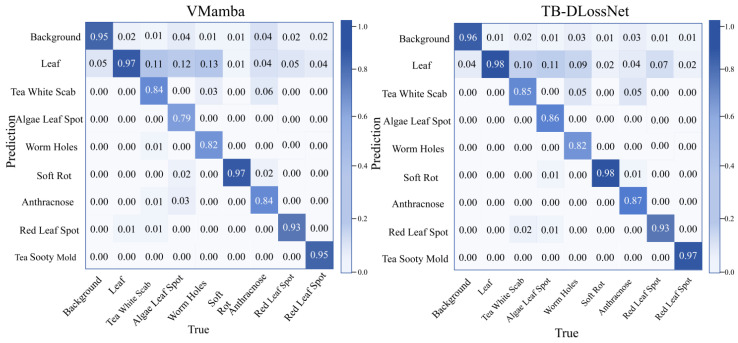
Comparative analysis of confusion matrices between the baseline and the optimized networks.

In order to ensure fair and standardized comparison, all models use the same data partition, input resolution and optimization scheme for training and evaluation. The performance evaluation is carried out on the public test set, and the standard indicators used include the average crossover ratio (mIoU), accuracy rate, recall rate and F1 score. Table 3 summarizes the quantitative comparison results, and Figure 1 presents the qualitative segmentation results of all models in a visual way.

As evidenced in Table 3, among the unimodal paradigms, VMamba establishes a robust baseline with an mIoU of 82.12% and an F1-score of 89.94%, outperforming most conventional CNN-based architectures. This underscores the potent representational capacity of state-space-based backbones for agricultural diagnostic tasks. The inclusion of textual priors in LViT further elevates the mIoU to 84.43%, validating the beneficial impact of semantic conditioning on pixel-level decision-making. In comparison, the proposed TB-DLossNet achieves state-of-the-art (SOTA) performance across all four metrics (mIoU: 87.02%, Precision: 93.75%, Recall: 92.10%, F1: 92.89%). Specifically, the substantial gain in mIoU reflects superior spatial overlap fidelity and boundary alignment. Notably, the concurrent optimization of Precision and Recall indicates that TB-DLossNet effectively suppresses both false positives and false negatives, leading to a markedly improved F1-score. Compared to LViT, TB-DLossNet exhibits improvements of 2.59% in mIoU and 1.50% in F1-score; relative to the unimodal VMamba, these gains reach 4.9% and 2.95%, respectively. These results demonstrate that our task-specific adaptations to the VMamba backbone not only enhance overall segmentation quality but also achieve a superior equilibrium in the precision-recall trade-off.

Although OneFormer and Mask2Former represent advanced query-based transformer segmentation frameworks, their performance on the proposed agricultural disease dataset is inferior to TB-DLossNet. This phenomenon can be attributed to several factors. Transformer-based query segmentation models are typically optimized for large-scale natural image benchmarks, where abundant training data facilitates the learning of global contextual representations and stable bipartite matching mechanisms. In the present study, however, the dataset comprises only 1400 images across nine fine-grained categories, which constrains the capacity of large transformer architectures to fully leverage their modeling potential. Plant disease segmentation further presents domain-specific challenges, including small lesion regions, irregular boundaries, high intra-class similarity, and considerable background interference. Under such conditions, architectures that primarily rely on global self-attention may struggle to adequately capture boundary-sensitive and fine-grained local discriminative features. By contrast, TB-DLossNet incorporates multimodal conditioning and task-oriented boundary enhancement, thereby embedding stronger task-specific inductive biases that are better aligned with the characteristics of agricultural disease segmentation.

VMamba is adopted as the visual backbone in this work due to its state-space modeling mechanism, which offers key advantages for fine-grained disease segmentation. Unlike Transformer-based architectures whose self-attention scales quadratically with image resolution, VMamba maintains linear computational complexity, enabling processing of high-resolution leaf images while preserving small lesion details that might otherwise be lost through downsampling. Its selective scanning mechanism traverses image patches along multiple spatial directions, capturing global contextual relationships across the entire leaf while maintaining local boundary precision. This combination of global context and local detail preservation is particularly beneficial for agricultural disease segmentation, where lesions often exhibit subtle boundaries, irregular shapes, and diverse spatial distributions. The empirical results in Table 3 support this theoretical basis: in the baseline model based solely on visual features, the VMamba-based model achieved the highest mIoU (82.12%), outperforming other representative architectures.

While the aggregate mIoU provides a holistic performance indicator, it may fail to capture the nuances of field-acquired *Camellia oleifera* imagery, where lesion categories exhibit high variance in frequency, scale, and morphology. The single average may be affected by the main background pixels or easily recognized categories and produce deviations, thus covering up the inherent difficulties of dividing key lesions. To address this, we provide a granular class-wise IoU analysis in Table 4. This enables a rigorous comparison of the strengths and limitations of each method across specific pathologies, establishing a factual basis for analyzing error patterns and assessing the reliability of the proposed framework for practical agronomic deployment.

The comparison table given in Table 4 shows that the disparity between performances across the seven lesion types is significant, with a similar pattern emerging: large-scale and highly visible pathologies are easily separable, but fragmented or small scale lesions remain problematic. Example: Soft rot and Sooty mold demonstrate high IoU scores across almost all the models examined. Conversely, categories like White scab and Insects-induced holes indicate significant performance differentials between the two methods being compared. Figure 1 presents the confusion matrices for both the baseline VMamba and the proposed TB-DLossNet, where diagonal entries represent categorical accuracy. Compared with the baseline, the proposed model achieves higher diagonal values across most disease categories. Notably, Algal Leaf Spot recognition accuracy increases from 79% to 86%, while categories such as White Scab and Worm Holes also show clear improvements. These gains are particularly meaningful for lesion types that are smaller or more fragmented, where the baseline struggled. Overall, the consistent accuracy improvements across multiple categories confirm the effectiveness of the proposed architectural enhancements.

Quantitative analysis shows that TB-DLossNet reaches a 2.96 higher mean IoU score (84.68%) on seven lesion categories compared to the runner up model, LViT (81.72%), and has the highest rank among numerous models. It becomes especially evident in the pathologies requiring fine-grained texture differentiation and boundary constancy (e.g., White scab, Algal leaf spot, and Anthracnose). Remarkably, for algal leaf spot disease, the performance of the proposed model significantly outperforms other models, achieving a 5.31% higher accuracy compared to the next best model. Our findings highlight the power of our framework to discrete lesions shapes, large scale diversity, and background-texture uncertainty. TB-DLossNet achieves high fidelity in spatial overlap of the true lesions with good suppression of false positive by improving segmentation quality of the true lesions, and thus performs better with fragmented lesions and multi-scale pathological characteristics.

It is worth noting that the dataset exhibits a certain degree of class imbalance. For instance, the Tea White Scab category contains 554 samples, whereas the Anthracnose category includes only 134 samples. Despite this imbalance, the proposed method maintains strong segmentation performance on minority classes. As shown in Table 4, our approach achieves an IoU of 82.93% on the Anthracnose category, outperforming all compared baseline methods. This result demonstrates that the proposed multimodal framework can still learn effective representations even when training samples are limited. We attribute this robustness to the combination of the dynamic loss mechanism and textual semantic priors, which help the model capture disease-related semantic cues and improve feature learning for underrepresented categories.

**Figure 2:**
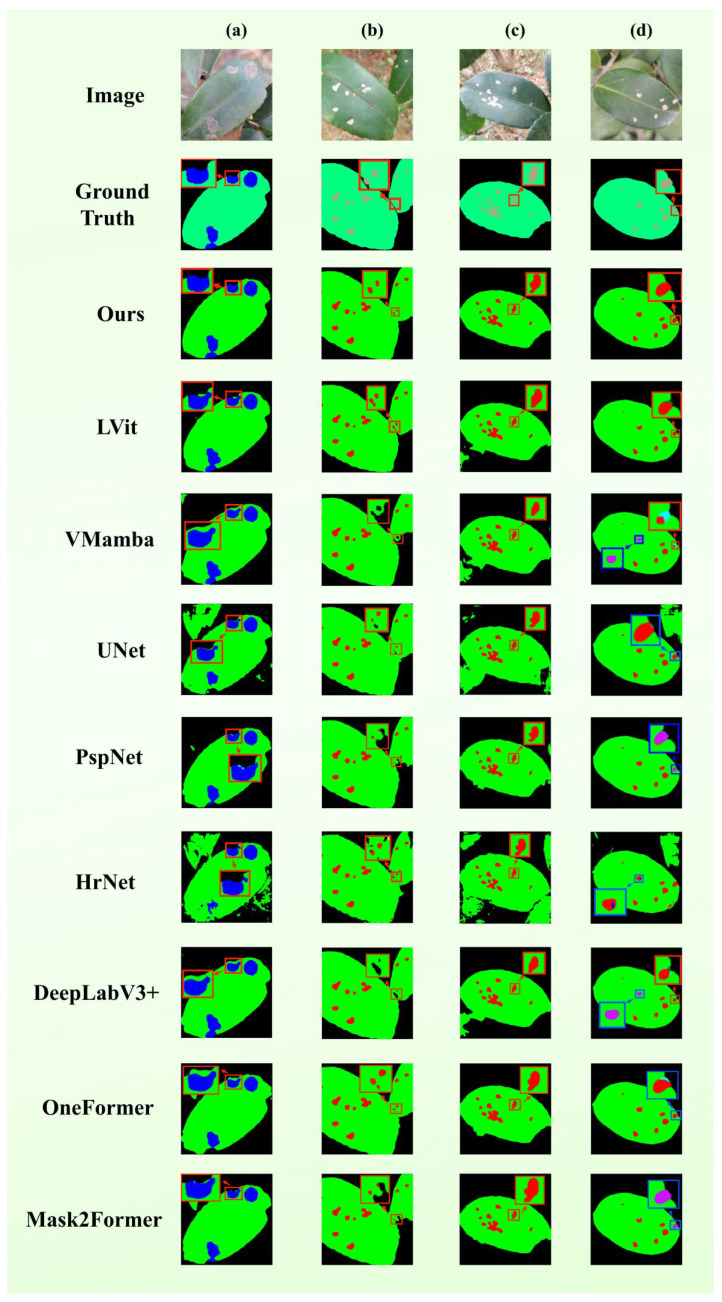
Qualitative segmentation comparison of various models on typical field specimens. Each panel illustrates original images, Ground Truth (GT), and corresponding predictions across models. Blue boxes denote instances of semantic misclassification. Scenarios include: (**a**) Algae leaf spot, (**b**) multi-leaf overlap, (**c**) leaf occlusion, and (**d**) sparse lesion distributions.

Figure 2 provides a qualitative comparison between TB-DLossNet and several representative baselines on the test set. Each row presents a typical field specimen, displaying (from left to right) the original image, ground truth, and the predicted masks of various models. In these visualizations, black denotes the background, green identifies the leaf tissue, and other colors differentiate disease categories. Observations from the representative cases include: (a) Algal leaf spot: TB-DLossNet exhibits more concentrated and precise localization that aligns closely with the ground truth, effectively avoiding the misclassification of foliar textures as lesions. (b) Multi-leaf scenarios: While several baselines tend to misidentify inter-leaf gaps as foliar tissue, TB-DLossNet accurately demarcates leaf boundaries, ensuring stable and complete lesion detection. (c) Occluded specimens: Unlike competing methods that suffer from fragmented predictions or background noise at leaf margins, our model demonstrates lower sensitivity to environmental artifacts and maintains superior structural coherence. (d) Sparse lesions: TB-DLossNet significantly reduces false positives and inter-class confusion, yielding predictions highly consistent with expert annotations.

Collectively, these qualitative evaluations confirm that the proposed framework successfully reconciles global semantic consistency of the leaf canopy with fine-grained recognition of pathological regions, reinforcing the quantitative trends observed in the class-wise IoU analysis.

### 3.3. Experiments on the Effectiveness of Multi-Layer Supervision

Multi-layer (deep) supervision promotes robust feature learning by introducing side output layers and auxiliary losses in the middle stage of the network. This architecture design ensures that the supervision signal is not limited to the final output, but directly constrains the feature representation at all levels, thus alleviating the problems of insufficient supervision and hindered gradient propagation inherent in the deep architecture. Considering the differences in semantic abstraction and spatial resolution levels of features of different depths, the placement of auxiliary supervision significantly affects the convergence and final performance of the model. Therefore, it is crucial to conduct an empirical assessment to determine the optimal configuration of these regulatory levels.

To this end, we performed a comparative analysis by varying the layer-wise configurations of side-output supervision while maintaining constant network hyperparameters and training protocols. These results are compared against a baseline model devoid of deep supervision, as detailed in Table 5. Drawing upon the findings of [44], which highlight that early hidden layers often lack sufficient discriminative power and exhibit semantic opacity, we strategically restricted the application of deep supervision to the intermediate and high-level layers of the encoder-decoder framework.

The empirical findings prove that the integrated implementation of the deep supervision of the all three layers 2, 3 and 4 is the best performer. The given statement underlines the fact that effectiveness of multi-layer monitoring depends on the strategic correspondence between hierarchical features, as well as their synergy. Namely, constraining separate or limited layers normally leads to suboptimal or partial optimization. As an example, supervising the middle states (the two layers numbered 2 and 3) focuses on the local structural aspects at the cost of the overall semantic guidance that may cause a destabilization in semantic class identification. Alternatively, concentrating on the deeper layers (the layers numbered 3 and 4) offers semantic constraints only but no supplemental control signals due to initial layers, which cannot properly optimize finer details or guarantee stable optimization.

Conversely, the joint supervision of layers 2, 3, and 4 effectively reconciles mid-level geometric structures with high-level semantics. This setup will deliver better results in all aggregate performance measures because it allows a more dense and efficient gradient flow across the entire architecture during training.

### 3.4. Ablations

To quantitatively assess the individual contributions of the proposed modules to segmentation performance, we conducted a series of ablation studies under a standardized training configuration. To maintain experimental rigor, all variables except the module under evaluation remained constant. Given the extensive permutations of component combinations, the findings are categorized into standalone module gains and synergistic effects, as summarized in Table 6 and Table 7, respectively.

The Table 6 displays the quantitative results of the baseline model as well as four stand-alone module setups. There is an indication indicating that all components have a performance yield, which is based on different types of analysis. It is worth noting that the addition of the Text module is the most significant improvement because there is not only an increase of the mIoU (82.12–85.82) but also significant improvements of the Recall and F1-score. It tells us that textual conditioning improves model detection completeness and spatial overlap fidelity substantially. Moreover, Precision metric stability ensures that such benefits can be not attributed to more conservative prediction threshold, but to enhanced feature matching and exhaustiveness.

To understand how textual priors enhance semantic discrimination, particularly for visually similar lesions, we conducted a detailed analysis at both feature and prediction levels. Visual-only models often produce overlapping feature representations for lesions with similar color or texture, such as Anthracnose and Red Leaf Spot, leading to misclassification. Incorporating textual embeddings introduces an additional semantic axis in the intermediate feature space, effectively increasing inter-class separability and making visually similar lesions more distinguishable. These embeddings also act as conditioning signals that modulate intermediate feature maps, amplifying features corresponding to textual descriptors and suppressing irrelevant patterns, allowing the network to focus on subtle spatial cues that are otherwise difficult to capture. Furthermore, textual descriptions encode human knowledge about lesion morphology, color, and distribution, which regularizes the prediction space and discourages semantically implausible segmentations, especially for rare or subtle lesions.

Exceeding textual modality, the Dyn, Boundary, and DS modules are of a more specialized gain profile. The Dyn (Dynamic Loss) module is always improving with an increase of 1.42% on mIoU and 0.75% on F1-score respectively, which confirms its effectiveness in influencing what the optimization is focused on within the dataset. When operating independently, however, Boundary and DS (Deep Supervision) only provide small gains in mIoU (Boundary: 0.12% and DS: 0.28%). It means that these components play the major part in smoothing out structural prediction and optimizing the flow of it. Namely, the Boundary module ensures critical geometric restrictions, and DS enhances the representational stability of the centralised latent representations.

To sum up, individual assessments in Table 6 confirm the effectiveness of the suggested element. These results imply that the high-leveled semantic conditioning (Text), geometric structure limitations (Boundary/DS), and dynamic training plans (Dyn) are very complementary, which creates solid grounds to implement the synergy of these three factors to obtain a better aggregate outcome.

The combination of the proposed modules To explore the synergistic relationships between the suggested components, a summary of the performance of several module combinations incorporated in the baseline architecture is presented in Table 7. Examples of this category are the text-conditioned guidance branch, the boundary enhancement module, multi-level side-output supervision (DS) and the dynamic loss weighting strategy. The purpose of this analysis is to determine if such components demonstrate cross-functional complementarity and thus provide cumulative performance improvements over their individual performances.

The combined use of the boundary enhancement branch and multi-level deep supervision along with the text-conditioned branch lead to an mIoU of 85.82% (+3.70% over the baseline). This configuration is also higher than the text-only in terms of spatial overlap fidelity and aggregate measures indicating the complementary nature of the high-level semantic constraint due to textual modality and the geometric refinement due to the structural branches during optimization. Likewise, adding dynamic re-weighting to the baseline + text combination will yield an mIoU of 86.33% (i.e., 4.21 higher than the baseline), which indicates that adaptive weights can offer long-term benefits to performance regardless of the semantic priors that are already present.

The best performance is achieved when all four components are activated simultaneously, giving an mIoU score of 87.02, Precision—93.75, and Recall—92.10, and F1-Score of 92.89. These findings suggest that the combined combination of various modules do not only support additive benefits but also guarantee a better balance between Precision and Recall to achieve an optimal level of total segmentation accuracy. To be more precise, semantic conditioning, structural constraints and adaptive training strategies together in one framework are expressed through simultaneous increase in both mIoU and F1-score. Additionally, boundary improvement combined with deep supervision has no textual input (ablation) which achieves an mIoU of 83.02 with an additional dynamic loss weighting that raises it to 84.15. It indicates that the dynamic weighting mechanism can deliver significant overlap gains regardless of multimodal priors.

### 3.5. Coexistence Segmentation Experiment for Multiple Disease Spots

**Figure 3:**
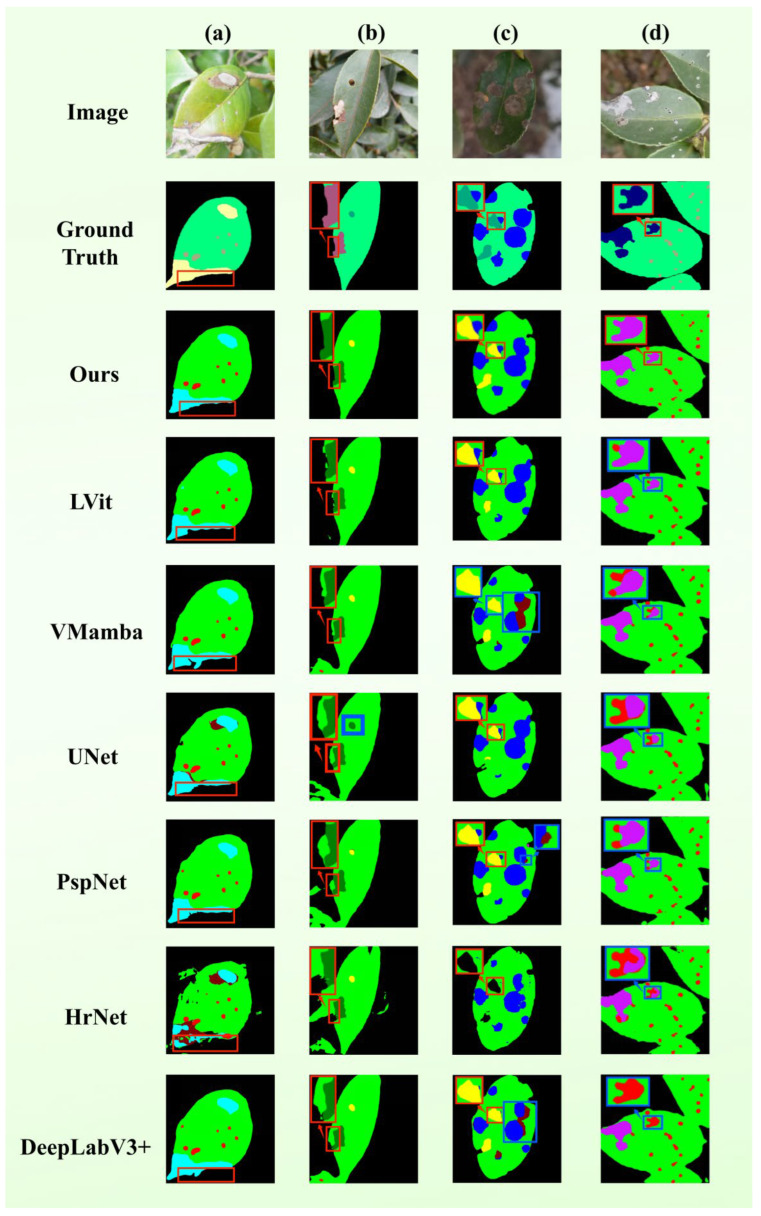
Comparative evaluation in multi-lesion coexistence scenarios. The figure contrasts original images and annotations with model predictions, where blue boxes highlight misclassified pathological categories. Cases shown are: (**a**) Multi-scale lesions, (**b**) lesions at foliar margins, (**c**) dense lesion clusters, and (**d**) interference from multi-leaf clutter.

To evaluate how well the framework works in cases where there are concurrent pathologies, we did a qualitative comparison through five test images, which had multiple lesions as shown in Figure 3. Multi-lesion environments, unlike solitary lesioned specimens, usually demonstrate higher lesion density rates, large size variability, and closer proximity between instances. The models need to recognize individual pathological areas in such situations and must be able to distinguish neighboring areas properly to prevent incorrect combination or omission of minor characteristics. Quantitative findings indicate that available comparative approaches often fall into the trap of semantic error and excessive omission rate in such intricate conditions.

The findings of the representative cases are as follows: (a) Multi-scale Lesions: TB-DLossNet was the only model to perform correct localization and classification. On the other hand, although the competing models recognized some areas, they were often ambiguous and omitted other areas. It is important to note that U-Net and HRNet had severe semantics misclassification with various lesion types. (b) Lesions at Foliar Margins: Most of the models (except U-Net) did classify the lesions correctly but TB-DLossNet alone successfully demarcated the boundaries of foliar margins which is necessary to achieve the boundary fidelity. (c) Dense Lesions Clusters: Due to the proximity of many lesions, only TB-DLossNet solved everything without omission. The competing frameworks recorded heavy false negatives and the models VMamba and DeepLabV3+ also mistook pathological classifications. (d) Interference from Multi-leaf Clutter: TB-DLossNet was the only model able to continue the same category recognition in presence of visual junk caused by overlapping leaves.

These results indicate that TB-DLossNet greatly reduces semantic confusion and omissions in multi-lesion settings. Also, the model strengthens the accuracy of boundary localization and guarantees structural integrity to enable more consistent and high-fidelity segmentation performance under diverse field conditions.

### 3.6. Small Lesion Segmentation Experiment

**Figure 4:**
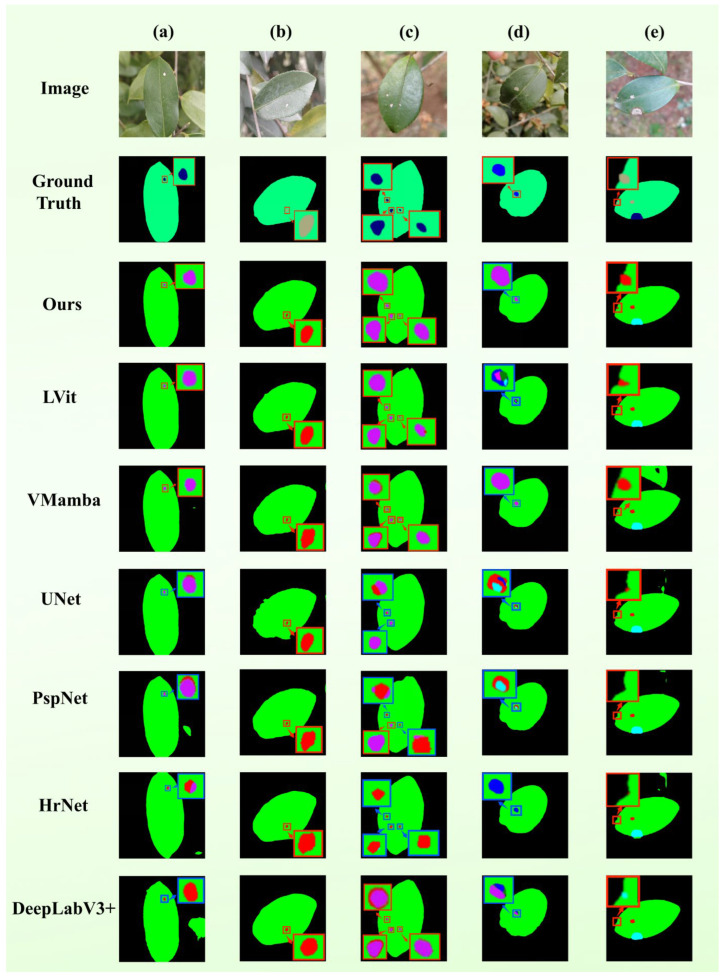
Segmentation performance for micro-scale lesions. Visualizations include original images, GT, and 4× localized magnifications of model outputs. Blue boxes identify categorical errors. Scenarios are categorized as: (**a**) Clean background, (**b**) distraction from foliar textures, (**c**) multi-target small lesions, (**d**) dense foliage, and (**e**) background-lesion ambiguity.

We have chosen five *Camellia oleifera* leaf pictures with minor lesions (less than 5-pixel occupancy) to compare qualitatively to evaluate the ability of the model to capture micro-scale objects, as shown in Figure 4. On those specimens, the areas affected by pathology have limited space and mild visual characteristics that can hide the fact that they can be overridden by the prevailing leaf tissues. Comparative findings in this variety of situations could be summarized in the following way:

(a) Clean Background: Most of the baselines (e.g., DeepLabV3+, HRNet, and PSPNet) failed to accurately recognize the specific diseases despite the successful localization of lesions by most models. However, only TB-DLossNet, LViT, and VMamba were correctly able to make the semantic identification. (b) Distraction from Foliar Textures: Though all the frameworks localized the pathological zones, various models had the displacement of boundaries of leaves contours. Only the TB-DLossNet and LViT had the highest level of structural fidelity concerning the borders of the leaves. (c) Multi-target Small Lesions: The only model with a consistent ability to classify all of the micro-lesions and their corresponding categories was the TB-DLossNet, whereas the competing models had serious problems with false-positive and false-negative errors. (d) Dense Foliage: TB-DLossNet and VMamba were the only architectures that accurately classified the lesions and other architectures could not eliminate intra-class ambiguity in these crowded scenes. (e) Background-Lesion Ambiguity: Due to the large size of the lesions and their apparent visual affinity with the background, TB-DLossNet is the only model that has extracted the pathological properties.

Together, these qualitative evaluations indicate that TB-DLossNet greatly improves the strength of segmentations and the reliability of diagnoses when dealing with small lesion cases that are harder to diagnose, and it is also effective at reducing the threat of omission and misclassification.

We further evaluated the performance of each model on leaves with small lesions, and the results are summarized in Table 8. Overall, our method achieved the highest IoU values across all nine categories, demonstrating stable and comprehensive performance advantages. It significantly outperformed other methods in both large-area categories (Background, Leaf) and small-scale lesion categories.

Compared with the evaluated baseline methods, the superior performance of TB-DLossNet in micro-lesion segmentation stems from four synergistic innovations. First, the Mamba backbone enables efficient global context modeling with linear complexity, preventing small lesions from being overlooked due to insufficient spatial context. Second, multimodal fusion with BERT-based semantic priors resolves visual ambiguity when pathological signatures are subtle or indistinguishable from background. Third, the area-adaptive dynamic loss function amplifies gradient contributions of micro-scale features, effectively reducing false negatives under severe class imbalance. Fourth, boundary-aware refinement with multi-scale deep supervision preserves structural integrity and sharp contours while ensuring robust gradient flow.

**Figure 5:**
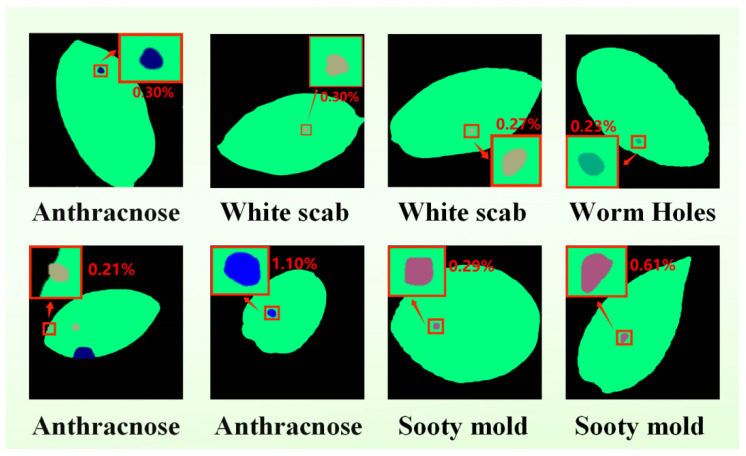
Quantitative assessment of lesion-to-leaf area ratios. Numerical annotations indicate the percentage occupancy of lesions relative to the leaf surface. Color coding represents: dark blue (Anthracnose), white (White scab), deep pink (Sooty mold), and dark green (Worm Holes).

To provide a rigorous characterization of small-scale infections, we quantitatively assessed the spatial extent of both pathological lesions and healthy foliar tissues. The resulting lesion-to-leaf area ratios are presented in Figure 5. The analysis encompasses four distinct pathologies: Anthracnose, White scab, Sooty mold, and Insect damage. Notably, the spatial occupancy of each disease category within the leaf area remains consistently below 2%, further validating the framework’s sensitivity and precision in detecting micro-scale pathological features.

### 3.7. Efficiency Evaluation

To evaluate the computational efficiency of the proposed model, we compared it with the original Vmamba in terms of number of parameters, FLOPs, and inference speed (FPS). The results are summarized in Table 9.

The total number of parameters in TB-DLossNet (162.8 million) includes the non-trainable frozen BERT text encoder (100 million). The actual trainable parameters amount to 62.8 million.

From Table 9, we observe that TB-DLossNet model has approximately 3.5 times the number of parameters compared to Vmamba, with a moderate increase in floating-point operations. This reflects the additional model capacity utilized to enhance feature representation and segmentation accuracy. Due to the increased size of TB-DLossNet, the frames per second (FPS) decreased from 35 to 22, indicating a trade-off between accuracy and speed. We acknowledge that in its current form, TB-DLossNet is not yet suitable for highly resource-constrained edge devices due to its model size and computational cost. However, the model achieves 22 FPS on GPU-enabled platforms, making it practical for near-real-time applications in scenarios where moderate computing resources are available, such as agricultural inspection systems equipped with GPU acceleration, cloud servers, or laboratory workstations.

In future work, we will pursue model compression to improve deployability on edge devices. Specifically, we plan to replace the BERT text encoder with a lightweight alternative such as DistilBERT, apply channel and layer pruning to reduce redundant structures, and adopt knowledge distillation to train a compact student model that retains segmentation accuracy.

### 3.8. Generalization Experiment

**Figure 6:**
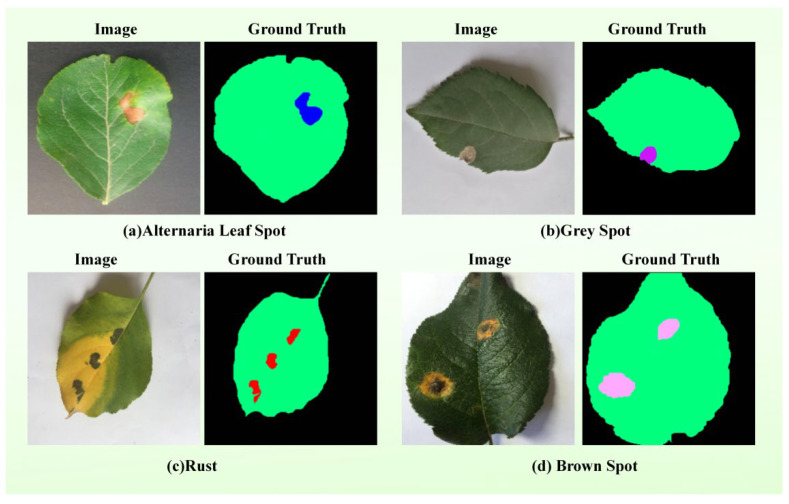
Sample images of the four disease categories included in the apple leaf disease dataset used in the generalization experiment: (**a**) Alternaria Leaf Spot, (**b**) Grey Spot, (**c**) Rust, (**d**) Brown Spot.

Given the scarcity of public multimodal agricultural segmentation datasets characterized by both complex natural backgrounds and aligned textual descriptions, we curated an external benchmark from the publicly available Apple dataset. The apple disease dataset used in this experiment contains 1000 images spanning four disease categories (Alternaria Leaf Spot, Grey Spot, Rust, Brown Spot). The text modality was not employed in this generalization experiment, as the publicly available dataset does not provide paired textual descriptions and the objective of the experiment was to evaluate the transferability of the vision-based components to unseen crop types. Samples were meticulously screened and taxonomically reorganized, involving label remapping into four distinct disease categories. This curated dataset serves as an independent benchmark to evaluate the model’s cross-species generalization performance. The example images of the four disease categories in this dataset are illustrated in Figure 6.

The performance of the proposed TB-DLossNet was benchmarked against the unimodal VMamba foundation across both datasets, with the comparative results detailed in Table 10.

Combining the empirical results of the two data sets, it can be clearly seen that TB-DLossNet has always been better than the baseline model VMamba in camellia and Apple benchmarks. This performance difference highlights the mobility of our proposed architecture improvement scheme for specific tasks. The cross-dataset evaluation on apple diseases confirms that TB-DLossNet generalizes across different crops, disease types, and imaging conditions. Despite domain shifts, our method consistently outperforms the baseline VMamba, demonstrating that the proposed architectural components learn transferable features rather than overfitting to the original training domain.

Although this model demonstrates superior segmentation performance compared to baseline methods, its relative advantage is smaller than that observed on the Camellia dataset. This performance gap can be attributed to domain difference effects. Specifically, apple leaf lesions differ from camellia leaf lesions in shape, size, color, and texture, reducing the effectiveness of the visual-textual prior learned on the camellia leaf lesion dataset. Additionally, variations in imaging conditions—including lighting, resolution, background, and shooting angle—impact feature extraction and segmentation accuracy. Differences in annotation standards, such as mask granularity or boundary precision, may further contribute to the observed discrepancies. Despite these factors, TB-DLossNet maintains robust segmentation performance across different lesion types, demonstrating strong generalization capabilities. Future work may explore domain adaptation or multi-domain training to further enhance robustness across datasets.

It is worth noting that the Apple dataset does not provide paired textual descriptions; therefore, the text branch was disabled in this experiment. Despite the absence of textual input, TB-DLossNet still consistently outperformed the baseline model, suggesting that the proposed framework does not rely on text as a strict prerequisite for segmentation. Instead, textual information mainly acts as an auxiliary semantic prior, while the visual branch preserves the core segmentation capability. Nevertheless, the current study does not systematically quantify the effect of noisy or ambiguous text, which will be explored in future work.

## 4. Conclusions

This research aims to solve the challenge of fine division of leaf diseases of *Camellia oleifera*, especially for the complex situations unique to the field, such as multi-target coexistence, significant scale differences and low occupancy rate of micro-disease spots. We proposed a model called TB-DLossNet (a text conditional boundary perception network based on dynamic loss reweighting), which optimizes the architecture of segmentation tasks on the VMamba visual state space (VSS) backbone network. Unlike the traditional single-modal architecture, TB-DLossNet integrates structured text a prior information into the visual coding stage. Through multi-level feature fusion, it imposes strict semantic constraints on pixel-level classification, which effectively alleviates semantic ambiguity in visual similar areas. At the same time, we have also introduced boundary perception branches and multi-scale in-depth supervision to make up for the structural shortcomings inherent in the region-based monitoring method. With the regional adaptive dynamic loss weighting strategy, the framework ensures that the continuous gradient focuses on small-scale pathological features, thus enhancing training stability and predictive consistency.

Experimental evaluations on our curated *Camellia oleifera* dataset demonstrate that TB-DLossNet significantly outperforms the VMamba baseline, with mIoU and F1-score increasing from 82.12% to 87.02% and 89.94% to 92.89%, respectively. These gains reflect a substantial enhancement in spatial overlap fidelity and categorical discrimination. Furthermore, benchmarking against several unimodal networks and multimodal state-of-the-art (SOTA) models confirms the superior efficacy of the proposed framework. Ablation studies elucidate the complementary roles of the integrated modules: textual conditioning refines semantic differentiation in cluttered environments; boundary enhancement alongside deep supervision preserves structural coherence and contour precision; and dynamic loss weighting amplifies the optimization focus on micro-lesions. The robust performance of TB-DLossNet on an external apple disease dataset further validates its transferability and generalization potential across diverse crop species.

In short, TB-DLossNet provides high-fidelity pixel-level segmentation, laying a solid foundation for downstream agricultural applications (including disease severity assessment and automated field management). Future research will focus on verifying the framework in a wider range of multi-crop data sets and different data collection environments. We also plan to explore more efficient text data collection strategies and finer boundary modeling techniques to further enhance the robustness of the model and the feasibility of deployment in actual agricultural scenarios.

We acknowledge that our current dataset is limited in scale, as large-scale multimodal datasets for agricultural lesion segmentation remain scarce. Future work will validate TB-DLossNet on emerging public datasets and explore domain adaptation to enhance generalization across diverse disease types. Concurrently, we will explicitly prioritize lightweighting as a core direction for future research to enable practical deployment of the model.

## 5. Materials and Methods

### 5.1. Data Collection

**Figure 7:**
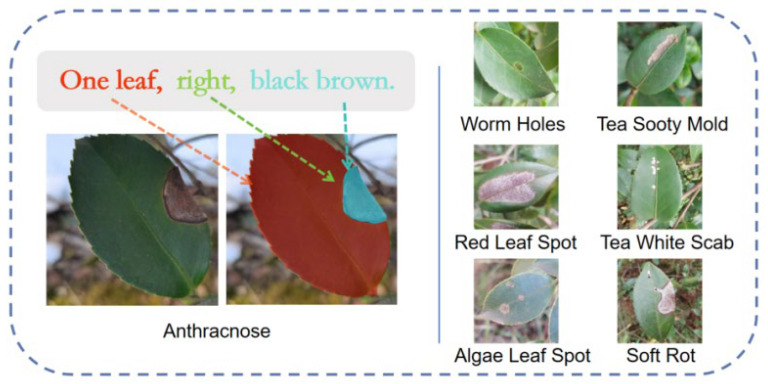
The textual annotations in the top-left corner correspond to the labels in the bottom panel, specifying the leaf count, lesion location, and lesion coloration, respectively. The central image illustrates Anthracnose. Displayed on the right are six additional pathologies.

The dataset curated for this research is a multimodal collection comprising aligned imagery and textual descriptors, as illustrated in Figure 7. The textual modality characterizes the number of leaves, the spatial distribution of lesions, and their specific coloration. These descriptions do not duplicate the pixel-level coordinate information in the mask annotations but instead provide complementary semantic guidance during feature learning. While mask labels supply explicit geometric supervision, textual prompts introduce high-level contextual information. Moreover, the prompts describe symptomatic characteristics rather than disease category labels, encouraging the model to focus on visual pathology features instead of class identifiers and thereby improving generalization. Integrating these semantic cues with visual data provides a holistic representation of *Camellia oleifera* pathologies, thereby facilitating the guided optimization of deep learning models for precision segmentation.

The proposed dataset comprises seven disease categories with varying numbers of annotated masks. The distribution of masks across categories is shown in Table 11. It is evident that the dataset is imbalanced, as Tea White Scab has over four times more instances than Anthracnose.

Image acquisition was conducted across *Camellia oleifera* plantations in Changde, Changsha, and Linli, Hunan Province, China, spanning from April 2023 to June 2025. The resulting dataset encompasses seven distinct pathological categories tailored for pixel-level segmentation tasks. All specimens were captured using a mobile imaging platform (iPhone 14) at a native resolution of 3456 × 3456 pixels, maintaining a working distance of 5–10 cm. To ensure robust generalization, images were acquired from diverse perspectives. For model training and evaluation, the data were randomly partitioned into training, validation, and testing sets with a ratio of 70:15:15, respectively. Prior to feature extraction, all images were standardized to a resolution of 512 × 512 pixels—a configuration chosen to balance computational efficiency with the preservation of micro-lesions and critical boundary details.

Image annotation was meticulously performed using a labeling platform integrated with the Segment Anything Model (SAM) [45]. The annotation protocol prioritized the high-precision segmentation of pathological lesions and healthy foliar tissues, adhering strictly to the Pascal VOC2007 metadata standard. Throughout the labeling process, particular emphasis was placed on boundary fidelity to ensure the model could effectively extract discriminative features from diseased regions.

It is important to clarify the distinction between SAM’s role as an interactive annotation tool and TB-DLossNet as an automated segmentation model. SAM is designed as a prompt-based foundation model requiring real-time human guidance (e.g., points or bounding boxes) at inference time, making it ideal for human-in-the-loop annotation but unsuitable for fully automatic deployment. In contrast, TB-DLossNet utilizes textual descriptions that are pre-annotated once during data preparation, not real-time user inputs. These descriptions provide stable task definition and continuous semantic guidance throughout feature extraction, enabling the model to address the specific challenges of agricultural lesion segmentation—subtle boundary ambiguity, small lesion areas, and severe pixel-level imbalance—that limit SAM’s direct applicability for fully automatic deployment.

To diversify the training distribution and bolster the model’s generalization capacity, a comprehensive suite of data augmentation strategies was applied to the training set. These encompassed geometric transformations (e.g., stochastic rotation, horizontal/vertical flipping, scaling, and random cropping), color jittering (adjusting brightness, contrast, and saturation), and Gaussian blurring. The intensity of color jittering and Gaussian blurring was carefully controlled to avoid excessive distortion of lesion regions, particularly for small lesions that occupy only a limited portion of the leaf surface. These augmentations primarily modify global appearance attributes, such as illumination and color distribution, while preserving the spatial structure and morphological characteristics of lesion areas. These techniques significantly enhanced the framework’s robustness across heterogeneous field environments while concurrently mitigating the risk of overfitting.

Although the proposed dataset contains 1400 images, it covers multiple disease categories collected under diverse field conditions. We adopt a stratified train/validation/test split and evaluate against several representative segmentation baselines, where consistent improvements are observed. Nevertheless, we acknowledge that a larger dataset and k-fold cross-validation could further strengthen statistical reliability and generalization assessment, which we leave for future work.

### 5.2. Deep Learning Method

**Figure 8:**
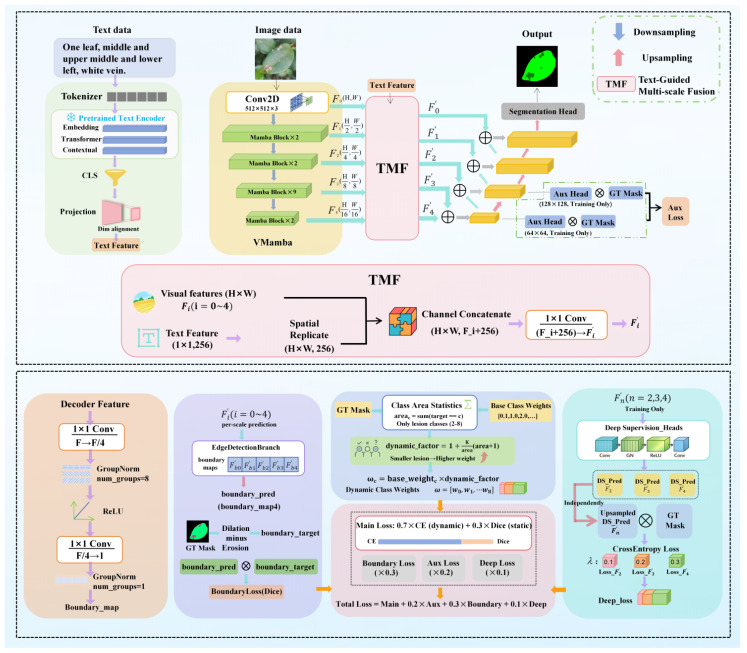
TB-DLossNet Overall Framework.

To enhance fine-grained segmentation precision and training stability for leaf disease imagery under challenging acquisition conditions, we implemented architectural adaptations to the VMamba network. Specifically, we propose a multimodal segmentation framework that synergistically integrates textual conditioning, Boundary-awareness, and Dynamic reweighting. Furthermore, multi-scale side-output supervision is incorporated during the training phase to stabilize the optimization process and reinforce the learning of intricate pathological details. This framework leverages the VMamba Visual State Space (VSS) encoder as its backbone, upon which task-specific extensions are constructed to facilitate pixel-level diagnostics. The comprehensive architecture of the proposed network is illustrated in Figure 8.

The lower part of Figure 8 illustrates the overall optimization framework, including the dynamic class weighting strategy, boundary-aware loss branch, and multi-scale deep supervision mechanism. Specifically, dynamic weights are computed based on lesion area statistics to alleviate class imbalance. The boundary branch generates refined edge maps using morphological operations (dilation and erosion) to enhance contour learning. In addition, deep supervision is applied to intermediate feature maps at multiple scales to stabilize training and improve convergence. The total loss is formulated as a weighted combination of the main loss, auxiliary loss, boundary loss, and deep supervision loss.

#### 5.2.1. Text Branch

Unimodal segmentation frameworks frequently necessitate autonomous semantic inference under challenging scenarios characterized by foliar leaf overlaps, stochastic lesion positioning, and chromatic evolution across pathological stages. Such conditions often precipitate localization offsets or background-lesion confusion. To provide the network with explicit global semantic priors, we introduce a textual branch that encodes image-aligned descriptions into global semantic vectors. These vectors are subsequently fused with multi-scale visual features to impose definitive semantic constraints. This paradigm aligns with recent advancements such as S-Seg [46] and DPSeg [47], which leverage open-vocabulary descriptors and CLIP-based embeddings, respectively, to refine pixel-wise predictions.

Specifically, we utilize a pre-trained BERT model [48] to derive sentence-level semantic representations. The implementation protocol is as follows:

1. Textual Tokenization: Descriptive metadata is processed by the BERT tokenizer into token sequences. These include [CLS] and [SEP] markers, truncated to a maximum length of 128 tokens, alongside the generation of corresponding token IDs and attention masks.
2. Semantic Feature Extraction: The pre-trained BERT architecture processes the sequences, where the 768-dimensional embedding of the [CLS] token from the final hidden layer is extracted as the holistic semantic representation of the image.
3. Cross-Modality Alignment: A fully connected (FC) layer reduces the BERT embedding to 256 dimensions. This is followed by ReLU activation and LayerNorm to ensure dimensional and distributional alignment with the visual feature space.
4. Multimodal Integration: The aligned textual features are spatially replicated and concatenated with visual features along the channel dimension. A 1 × 1 convolution is then applied to modulate the channel depth, yielding a unified multimodal input for the VMamba encoder.

Through this architecture, the textual branch provides high-level semantic priors, enabling the framework to maintain superior semantic consistency and cross-sample robustness despite complex backgrounds and significant visual variations.

Although the proposed framework demonstrates that incorporating textual semantic priors can effectively improve lesion segmentation performance, the current study does not systematically investigate the influence of different forms of textual descriptions. In practical agricultural scenarios, expert-authored descriptions of the same disease may vary in information density, ranging from concise disease names to detailed morphological characteristics. Variations in textual specificity may influence the strength of cross-modal alignment between visual and textual features, potentially affecting the extent to which textual guidance benefits the segmentation model. Exploring how different levels of textual detail impact multimodal learning would therefore be an interesting direction for future research. Such analysis could further clarify the role of semantic priors in guiding visual feature learning for agricultural disease segmentation.

#### 5.2.2. Boundary Enhancement and Multi-Layer Supervision

To bolster the structural fidelity of segmentation results and enhance the convergence stability of the training process, we incorporate a boundary-aware multi-scale deep supervision (DS) mechanism parallel to the primary segmentation stream. This dual-purpose design serves two functions: first, it introduces a lightweight boundary prediction branch into the encoder’s multi-scale features to refine geometric representations via explicit boundary constraints; second, it implements auxiliary supervision across multiple levels of the encoder-decoder architecture. This ensures that intermediate layers receive direct optimization signals, thereby mitigating suboptimal gradient flow and the degradation of detail learning inherent in deep architectures that rely solely on final-stage backpropagation.

Architecturally, the boundary branch interfaces with the multimodal fused features at each scale. It employs a streamlined boundary prediction head—comprising two 1×1 convolutional layers interleaved with normalization and non-linear activation—to generate a single-channel boundary probability map, denoted as $\hat{B}\in {\left[0,1\right]}^{H\times W}$. To ensure spatial alignment for supervision, these probability maps are bilinearly upsampled to the ground-truth resolution during training. Notably, while the network is capable of generating multi-scale boundary maps, we supervise only the final-layer output to minimize hyperparameter optimization overhead and simplify the loss function.

In the absence of manual boundary annotations, the reference boundary B is derived automatically from the segmentation masks Y. Specifically, we apply morphological dilation and erosion operations to each category mask; the boundary zone is subsequently extracted as the element-wise difference between the dilated and eroded results:

(8) B=|Dilate(Y)−Erode(Y)|

Subsequently, a Dice-based boundary loss is employed to constrain the predicted boundaries and boundary supervision:

(9) $$L_{bd}=1-\frac{2\sum (\hat{B}\cdot B)+\epsilon }{\sum \hat{B}+\sum B+\epsilon }$$

Here, ϵ is a small constant for numerical stability. The key motivation for including the boundary term is to extend the optimization objective by incorporating interface information, thereby mitigating the limitations of regional loss functions in cases of extreme class imbalance or inadequate modeling of boundary details. Previous work has shown that the boundary term can be combined with standard regional losses and provides complementary supervisory signals [49].

To implement multi-layer supervision, we introduce side outputs from two sources: auxiliary outputs from the decoder and deep-supervision outputs from the encoder. This design enhances feature learning across different network stages. Specifically, the decoder auxiliary branch produces auxiliary logits $logits\{{\hat{S}}_{j}^{aux}\}$ from intermediate decoder features. These logits are upsampled to the spatial resolution of the ground-truth labels, and a cross-entropy loss is computed, yielding an auxiliary loss term.

(10) $$L_{aux}=\frac{1}{M}\sum_{j=1}^{M}CE({\hat{S}}_{j}^{aux},Y)$$

In parallel, a deep supervision branch is applied to the encoder. At several encoder scales, it produces deep supervision $logits\{{\hat{S}}_{k}^{deep}\}$ directly from the fused features. These logits are combined via a weighted sum using coefficients $\alpha_{\kappa }$:

(11) $$L_{deep}=\sum_{k=1}^{K}\alpha_{k}CE({\hat{S}}_{k}^{deep},Y)$$

Consequently, the boundary constraint and the multi-layer side-output supervision are integrated into the total objective function as weighted terms:

(12) $$L=L_{main}+\lambda_{bd}L_{bd}+\lambda_{aux}L_{aux}+\lambda_{deep}L_{deep}$$

Here, $L_{main}$ denotes the primary segmentation loss (detailed in the next section), while $\lambda_{bd}$, $\lambda_{aux}$, and $\lambda_{deep}$ are weighting coefficients that balance the influence of the boundary loss and the multi-layer supervision losses. This design enables the model to be guided by explicit boundary constraints and to receive gradient signals from multiple scales during training. This leads to more stable optimization and final segmentation predictions that exhibit both regional consistency and sharp contours.

It is worth noting that the boundary enhancement branch is not designed as a generic edge detector that responds to all high-frequency gradients. Instead, it is supervised using lesion-specific contour annotations, which guide the branch to focus on pathological boundary characteristics. Environmental interference such as dust particles or shadows may produce local gradient responses; however, such artifacts typically exhibit fragmented or structurally inconsistent edge patterns compared with genuine lesions, which tend to form relatively continuous and spatially coherent contours within leaf tissue. Furthermore, the global context modeling capability of the VMamba encoder provides an additional structural constraint. Isolated or contextually inconsistent edge activations are less likely to be consistently reinforced during feature aggregation, as the selective scan mechanism integrates long-range spatial dependencies across the image. Therefore, the boundary branch enhances lesion-relevant structures rather than indiscriminately amplifying arbitrary edge patterns.

Although the proposed boundary enhancement mechanism is guided by lesion-specific supervision and global contextual modeling, it may still encounter difficulties under extreme conditions where environmental interference closely resembles lesion morphology. This limitation is inherent to data-driven learning frameworks and will be further explored in future work.

#### 5.2.3. Dynamic Loss Re-Weighting

To mitigate issues of pixel-wise class imbalance and the inadequate gradient contribution from small objects during training, we incorporate dynamic weight learning into the loss function. This dynamic loss component is integrated with the Dice loss (for regional consistency), the boundary constraint, and the multi-layer side-output supervision to form the complete objective function. The primary segmentation loss L_main is a weighted combination of cross-entropy and Dice:

(13) $$L_{main}=\lambda_{ce}L_{ce}^{dyn}+\lambda_{dice}L_{dice}$$

The key idea of the dynamic weighting scheme is to assign higher loss weights to classes with fewer pixels in the current batch, thereby amplifying their gradient influence. Formally, let the set of classes be {0, …, C − 1}. We first define a set of static prior weights $\omega_{c}^{base}$, where the background class is assigned a lower weight, the leaf class a medium weight, and the disease classes higher weights. Dynamic enhancement is then applied only to the subset of disease classes $C_{d}$. For a given disease class c within the current batch, its pixel count $A_{c}$ in the ground-truth label is calculated as:

(14) $$A_{c}=\sum_{p}I(Y_{p}=c)$$

where p indexes pixel locations, $Y_{p}$ is the ground-truth label, and I(·) is the indicator function. The dynamic enhancement factor for class c is subsequently defined as:

(15) $$g_{c}=1+\frac{K}{A_{c}+1},\;c\in C_{d}$$

Here, K is a scaling factor. This formulation ensures an inverse relationship between $A_{c}$ and $g_{c}$, thereby providing a stronger learning signal for small lesions. To prevent weight explosion caused by extreme batch statistics, we clamp the values of $A_{c}$ and $g_{c}$ and apply numerical stabilization to the final weights. The dynamically adjusted weight for class c, used in the cross-entropy loss, is:

(16) $$w_{c}^{dyn}=\{\begin{array}{ll} w_{c}^{base}\cdot g_{c}, & c\in C_{d},\\ w_{c}^{base}, & otherwise. \end{array}$$

This weight is incorporated into the weighted cross-entropy loss as follows:

(17) $$L_{ce}^{dyn}=-\sum_{p}w_{Y_{p}}^{dyn}\log P(Y_{p}|p)$$

To prevent the relative emphasis on disease classes from being diluted by a global normalization, we do not normalize weights across all classes. Instead, mean normalization is applied only within the set of disease classes $C_{d}$, preserving their internal weight ratios. This serves as a practical stabilization strategy for the segmentation task. Furthermore, to mitigate optimization bias stemming from the dominance of the background or other large classes, we employ the Dice loss as a regional overlap constraint. The Dice loss is effective in handling class imbalance, a property established in the V-Net framework [50].

## Figures and Tables

**Table 1:** Experimental Environment Configuration.

Environment	Device	Parameter
Hard ware environment	CPU	Intel(R) Xeon(R) Gold 5418Y
	GPU	NVIDIA GeForce RTX4090
	RAM	64 GB
	Video memory	24 GB
Soft ware environment	OS	Ubuntu 22.04.5 LTS
	CUDA Toolkit	12.1
	CUDNN	9.1.0.70
	Python	3.10.14
	Pytorch-GPU	2.5.0 + cu121
	torchvision	0.20.0 + cu121

**Table 2:** Model Training Hyperparameters.

Hyperparameters	Parameter
Image Size	512 × 512
Batch Size	4
Initial learning rate	0.0002
Optimizer	AdamW

**Table 3:** Performance Evaluation and Comparative Analysis of *Camellia oleifera* Disease Segmentation Frameworks. In this analysis, the symbols × and √ denote the exclusion and inclusion of textual features, respectively.

Method	Text	mIoU (%)	Precision (%)	Recall (%)	F1-Score (%)
VMamba	×	82.12	90.07	90.10	89.94
UNet	×	78.60	89.08	86.87	87.64
PSPNet	×	81.35	90.47	88.63	89.42
HRNet	×	73.56	85.41	83.02	83.89
DeepLabv3+	×	81.38	89.47	89.74	89.47
OneFormer	×	80.52	87.89	90.33	88.88
Mask2Former	×	81.32	89.75	89.30	89.37
LViT	√	84.43	91.86	91.04	91.39
TB-DLossNet	√	**87.02**	**93.75**	**92.10**	**92.89**

**Table 4:** Comparative Analysis of Class-wise IoU Performance for Evaluated Segmentation Frameworks. The best results for each category are in bold.

Category (IoU%)	Vmamba	UNet	PspNet	HrNet	DeeplabV3+	OneFormer	Mask2	Lvit	Ours
Background	94.19	89.46	94.58	84.13	94.12	93.81	94.95	95.34	**96.43**
Leaf	90.91	85.47	91.34	80.29	90.75	90.38	91.76	92.53	**93.99**
Tea White Scab	70.00	74.80	72.64	70.29	67.88	70.79	73.23	73.85	**77.5**
Algae Leaf Spot	74.32	73.08	71.17	67.66	77.10	69.25	69.20	79.24	**84.55**
Worm Holes	72.43	65.47	70.34	39.70	69.67	69.17	68.90	**75.84**	74.33
Soft Rot	86.40	90.43	87.59	89.14	82.62	85.94	86.54	84.25	**93.09**
Anthracnose	75.44	60.47	71.22	65.77	75.50	70.77	72.71	79.64	**82.93**
Red Leaf Spot	85.57	81.22	84.42	82.19	84.75	83.77	83.36	86.77	**87.29**
Tea Sooty Mold	89.83	86.96	88.84	82.84	90.05	90.85	91.25	92.44	**93.08**

**Table 5:** Effectiveness of multi-scale deep supervision configurations.

Supervision Level	mIoU (%)	Precision (%)	Recall (%)	F1-Score (%)
Without	82.12	90.07	90.10	89.94
L2	79.26	89.88	85.10	87.05
L3	81.29	90.19	86.66	88.14
L4	81.67	90.95	87.20	88.74
L2 + L3	81.37	90.36	86.63	88.20
L2 + L4	81.89	90.65	87.51	88.78
L3 + L4	81.97	90.36	86.63	88.20
L2 + L3 + L4	**82.62**	**92.62**	**88.41**	**90.21**

**Table 6:** Standalone contributions of individual modules. Note: The symbols × and √ denote the exclusion and inclusion of textual features, respectively. DS represents multi-scale deep supervision, and Dyn indicates the dynamic loss reweighting strategy.

Method	Text	mIoU (%)	Precision (%)	Recall (%)	F1-Score (%)
VMamba	×	82.12	90.07	90.10	89.94
VMamba	√	**85.82**	**91.65**	**92.41**	**91.94**
VMamba+Boundary	×	82.46	92.37	87.68	90.20
VMamba+DS	×	82.62	92.62	88.41	90.21
VMamba+Dyn	×	83.54	92.29	88.73	90.69

**Table 7:** Synergistic effects of module combinations. Note: The symbols × and √ denote the exclusion and inclusion of textual features, respectively. DS represents deep supervision, and Dyn indicates dynamic loss reweighting.

Method	Text	mIoU (%)	Precision (%)	Recall (%)	F1-Score (%)
VMamba+Boundary+DS	×	83.02	92.42	88.64	90.23
VMamba+Boundary+DS+Dyn	×	84.15	92.88	88.98	91.12
VMamba+Boundary+DS	√	86.54	93.22	92.02	92.52
VMamba+Dyn	√	86.33	93.20	91.85	92.43
VMamba+Boundary	√	85.91	92.88	91.26	92.06
VMamba+DS	√	86.01	93.03	91.58	92.30
VMamba+Boundary+DS+Dyn	√	**87.02**	**93.75**	**92.10**	**92.89**

**Table 8:** Performance Evaluation and Comparison of Small Lesion Datasets.

Category (IoU%)	Vmamba	UNet	PspNet	HrNet	DeeplabV3+	Lvit	Ours
Background	91.36	88.11	91.58	82.63	91.47	92.03	**92.94**
Leaf	86.48	83.36	86.54	78.58	86.74	87.54	**88.17**
Tea White Scab	44.58	42.22	43.63	40.17	47.85	50.41	**53.22**
Algae Leaf Spot	58.92	51.37	55.10	42.80	62.10	65.02	**69.44**
Worm Holes	34.77	32.88	32.74	30.35	32.60	37.95	**41.21**
Soft Rot	71.65	64.52	68.92	58.42	73.41	79.30	**83.12**
Anthracnose	58.43	50.74	56.41	49.73	60.52	65.12	**68.89**
Red Leaf Spot	66.21	58.83	64.28	56.64	69.53	74.08	**77.65**
Tea Sooty Mold	79.79	72.30	67.23	77.74	57.33	61.61	**82.94**

**Table 9:** Cost Analysis Calculation.

Mothed	Params	FLOPs	FPS
Vmamba	46.2 M	25 G	35
TB-DLossNet	62.8 M	32 G	22

**Table 10:** Cross-dataset performance evaluation and comparison.

Dataset	Mothed	mIoU (%)	Precision (%)	Recall (%)	F1-Score (%)
*Camellia oleifera*	Vmamba	82.12	90.07	90.10	89.94
*Camellia oleifera*	TB-DLossNet	**87.02**	**93.75**	**92.10**	**92.89**
Apple	Vmamba	82.87	88.53	91.97	89.63
Apple	TB-DLossNet	**86.74**	**91.11**	**92.39**	**91.72**

**Table 11:** Number of masks for various diseases.

Disease Category	Quantity	Proportion
Tea White Scab	554	42.1%
Algae Leaf Spot	203	15.4%
Worm Holes	238	18.1%
Soft Rot	203	15.4%
Anthracnose	134	10.2%
Red Leaf Spot	200	15.2%
Tea Sooty Mold	219	16.6%

## Data Availability

The data supporting the findings of this study are available from the corresponding author upon reasonable request. Our code and experimental dataset are available at https://github.com/zzzsq239/TB-1.
